# Lack of Association between Mannose Binding Lectin and Antibody Responses after Acellular Pertussis Vaccinations

**DOI:** 10.1371/journal.pone.0088919

**Published:** 2014-02-18

**Authors:** Kirsi Gröndahl-Yli-Hannuksela, Juho Vuononvirta, Ville Peltola, Jussi Mertsola, Qiushui He

**Affiliations:** 1 Department of Infectious Disease Surveillance and Control, National Institute for Health and Welfare (THL), Turku, Finland; 2 Department of Pediatrics, Turku University Hospital, Turku, Finland; University of Melbourne, Australia

## Abstract

**Background:**

Mannose-binding lectin (MBL) is one of the key molecules in innate immunity and its role in human vaccine responses is poorly known. This study aimed to investigate the possible association of MBL polymorphisms with antibody production after primary and booster vaccinations with acellular pertussis vaccines in infants and adolescents.

**Methodology/Principal Findings:**

Five hundred and sixty eight subjects were included in this study. In the adolescent cohort 355 subjects received a dose of diphtheria and tetanus toxoids and acellular pertussis (dTpa) vaccine ten years previously. Follow-up was performed at 3, 5 and 10 years. Infant cohort consisted of 213 subjects, who had received three primary doses of DTaP vaccine at 3, 5, and 12 months of age according to Finnish immunization program. Blood samples were collected before the vaccinations at 2,5 months of age and after the vaccinations at 13 months and 2 years of age. Concentrations of IgG antibodies to pertussis toxin, filamentous hemagglutinin, and pertactin and antibodies to diphtheria and tetanus toxoids were measured by standardized enzyme-linked immunosorbant assay. Single nucleotide polymorphisms of *MBL2* gene exon1 (codons 52, 54, 57) were examined. MBL serum concentration was also measured from the adolescent cohort. No association was found with MBL2 exon 1 polymorphisms and antibody responses against vaccine antigens, after primary and booster dTpa vaccination.

**Conclusions:**

This study indicates that MBL polymorphisms do not affect the production and persistence of antibodies after acellular pertussis vaccination. Our finding also suggests that MBL might not be involved in modulating antibody responses to the vaccines made of purified bacterial proteins.

## Introduction

Mannose-binding lectin (MBL) is an important, soluble pattern-recognition molecule of the innate immune system. MBL is able to recognize and bind to a wide variety of microbes, leading to activation of the lectin pathway of complement system. A wider role of MBL as a modulator of immune responses has been suggested recently, as MBL has been shown to interact with several targets such as altered self and immunoglobulins [1].

Pertussis is a vaccine preventable, respiratory tract infection mainly caused by *Bordetella pertussis.* Despite extensive childhood vaccinations in many developed countries, *B. pertussis* is still circulating and causing periodic outbreaks. Increasing incidence of pertussis is recognized especially in infants, adolescents and adults [2]–[4]. Immunity against pertussis, both after infection and immunization, is not life-long. Due to this, the identification of the immunological factors behind the protection against pertussis is one of the major goals in this area. In contrast to other vaccine-preventable diseases such as diphtheria or tetanus, where the protection is mediated by antibodies against one diseases causing toxin, *B. pertussis* produces various virulence factors such as toxins and adhesins, which play a crucial role in the pathogenesis of this disease [5]. Immunity induced by these multiple antigens makes the understanding of the protection mechanism more complicated. The level of Immunoglobulin G (IgG) antibodies against pertussis toxin, pertactin and fimbriae are though been correlated to the protection against pertussis [6], [7]. So far, the number of studies concerning innate immunity in protection against pertussis in humans is very limited.

Single nucleotide polymorphisms (SNPs) in the gene encoding MBL (*MBL2*) occur in codons 52 (allele D, no rs5030737), 54 (allele B, no rs1800450) and 57 (allele C, no rs1800451) [8]–[10]. These polymorphisms are known to cause reduced concentration of MBL in the blood [8], [11]. The deficiency of MBL, caused by the polymorphisms, has been associated with susceptibility to certain infectious diseases [12].

MBL has been shown to recognize many gram positive and gram negative bacteria, including *Haemophilus influenzae* which is a gram-negative bacterium [13]. *B. pertussis* is close to *H. influenza* and was called *Haemophilus pertussis* in 1950s. However, there is no *in vitro* or *in vivo* study showing whether MBL recognizes *B. pertussis* or not.

The knowledge of possible association of MBL on vaccine induced immune responses is minor. In humans, relationship between MBL polymorphism and poor antibody responses has been reported with inactivated influenza vaccine [14]. Other studies are from animal models, investigating group B Streptococcus, tetanus toxoid and viral vaccines [15], [16].

In humans, the number of clinical studies concerning innate immunity in protection against pertussis is low. To our knowledge, previous studies have only concentrated on polymorphisms in toll-like receptor 4 pathway and antibody responses to pertussis vaccines [17], [18]. It is not known whether MBL plays a role in modulating antibody responses after pertussis vaccination. However, our previous study indicated that MBL deficiency might increase the risk for pertussis in adults [19]. Although many clinical studies have shown that MBL deficiency is associated with susceptibilities to infections [20], no clinical study has been conducted to investigate any effect of MBL deficiency in vaccine responses. In this study, we compared IgG antibody responses after DTaP vaccination in Finnish infants and adolescents with MBL polymorphisms in codons 52, 54 and 57. The serum MBL concentrations from adolescent cohort were measured as well.

## Materials and Methods

### Study design and the study subjects

The study protocol was approved by the joint commission on ethics of the Turku University and the Turku University Central Hospital and written informed consent was obtained from the study subject prior to enrolment. The study was conducted in accordance with Good Clinical Practice Guidelines and the Somerset West, 1996 version of the Declaration of Helsinki. Written, informed consent was obtained from the parents or guardian of all children before their enrollment to the study.

### Adolescent cohort

The initial study started in 1997, Turku, Finland. Five hundred and ten adolescents, aged 11–13 years, were recruited to receive a single booster dose of acellular pertussis vaccine. A subgroup of 450 subjects received a single dose of dTpa vaccine (Boostrix, GlaxoSmithKline) and 60 subjects received a diphtheria and tetanus toxoid (dT) vaccine followed with acellular pertussis (ap) vaccine one month after [21]. A follow-up of the same cohort was conducted 3, 5 and 10 years after the booster vaccination [22]–[24]. At the 10-year follow-up, the same cohort was invited to receive an additional dTpa booster vaccine and 82 subjects enrolled in the study. The results of immunogenicity and reactogenicity of the second dTpa booster given at ten year follow-up have been published earlier [24]. For this study, a total of 355 subjects (206 female, 149 male) whose genomic DNA samples were available, were included. All subjects were white caucasians.

All subjects had received four doses of whole cell pertussis vaccine (DTwP) in childhood at the ages of 3, 4, 5 and 24 months. Whole cell pertussis vaccine was used until 2005, when acellular pertussis vaccine was introduced. The only pertussis booster vaccine used in Finland is Boostrix® (GlaxoSmithKline).

### Infant cohort

The infant study cohort included Finnish children who participated to the prospective cohort study called Steps to Children's Healthy Development and Wellbeing (STEPS) [25]. The STEPS-study included approximately 1,800 children who are followed-up from birth. For this study, 213 subjects with available DNA samples were selected. Serum samples were also collected from these healthy infants who visited the study clinic at the age of 2,5 months, 13 months and 2 years. Two and half month visit was conducted before the vaccinations according to the national vaccine program at 3 months. At 13 months of age all study subjects had received three doses of primary pertussis vaccinations according to the national vaccine program. For this study, subjects with available serum samples at the age of 2,5 months, 13 months and 2 years were included, which counts for 72, 213 and 108 subjects (108 male, 105 female), respectively. All subjects were white Caucasian. All subjects had received three doses of Finnish DTaP vaccine at the ages of 3, 5, and 12 months.

### Genotyping

In the adolescent cohort, from 75 subjects enrolled in the 10-year follow-up, genomic DNA was isolated from the blood with QiAmp DNA Blood Mini Kit 250 (Qiagen, Germany) [18]. From original study in 1997, we randomly selected 280 subjects whose sera were enough for DNA isolation. The DNA isolation was made by using NucleoSpin Plasma XS kit (Macherey-Nagel GmbH&Co, Germany). In infant cohort, genomic DNA was isolated from 213 subjects as described earlier [26]. Genotyping of the three *MBL2* polymorphism sites on exon1; codons 52, 54 and 57, were done with single pyrosequencing reaction. Before pyrosequencing, PCR reaction and the cycles were performed as described by Roos et al. (2006) [27]. The PCR product was verified with agarose electrophoresis, the specific band for MBL was 240 bp. Pyrosequencing of *MBL2* was performed as described previously [27] with minor modifications described earlier [18]. The MBL genotypes were categorized as A/A as the wild type, heterozygotes variants as A/O and homozygotes variants as O/O. O stands for any variant alleles B, C or D.

### Antigen specific antibody measurement

From the adolescent cohort, IgG antibodies to all vaccine antigens: pertussis toxin (PT), filamentous hemagglutinin (FHA), pertactin (PRN), diphtheria (D) and tetanus toxoids (T) were measured earlier using ELISA, and the results have been published [21]–[24]. The detection limit for pertussis antibodies was 5 IU/ml, and for the diphtheria and tetanus antibodies 0,1 IU/ml.

From the infant cohort, IgG antibodies against PT, FHA and PRN were measured from available serum samples at age of 2,5 months, 13 months and 2 years, which counts for 72, 213 and 108 serum samples, respectively. Pertussis toxin IgG measurement was performed with ELISA method in which pertussis toxin is used as a coating molecule, as described previously [28] with minor modifications [29]. FHA and PRN IgG measurements were also performed with ELISA method. For coating, 2 µg/ml of purified FHA or PRN antigen was used. All the three antigens were kindly provided by GlaxoSmithKline, Belgium. From each serum sample, four dilutions were included (1∶60; 1∶240; 1∶960 and 1∶3840). For creating the standard curve, commercial Pertussis Antiserum (human) 1st IS - WHO international Standard serum (06/142, NIBSC, UK) was used with eight dilutions (1∶60; 1∶120; 1∶240; 1∶480; 1∶960; 1∶1920; 1∶3840; 1∶7680). The concentration of FHA and PRN IgG antibodies in the standard serum is 122 IU/ml and 39 IU/ml, respectively [30]. As secondary antibodies, ReserveAP anti-human IgG (gamma) phosphatase labelled antibodies were used (Kirkegaard & Perry Laboratories, catno. 0751-1002). The absorbance was read at 405 nm. Positive and negative in-house controls were included in each run. The concentrations (international unit/ml) of anti-PT, anti-FHA and anti-PRN were calculated with UnitCalc-software (Sweden).

For statistical analysis, subjects with undetectable concentration of IgG antibodies were given a value 0.1 IU/ml.

### Serum MBL concentration measurement

The serum MBL concentration was measured using double-antibody sandwich ELISA as described previously [19]. The detection limit for the assay is 50 ng/ml. For statistical analysis, results below the cut-off value were given a value 25 ng/ml.

### Statistical analysis

The Mann-Whitney U test, Kruskal-Wallis 1-way ANOVA and χ2 or Fishers' exact tests were used to identify differences between genotypes. These statistical tests were two-tailed, and a p-value of <0.05 was regarded as significant. Statistical analyses were performed with Prism GraphPad version 4.0 (GraphPad Software Inc., USA).

## Results

### MBL genotypes and concentration

**Figure 1:**
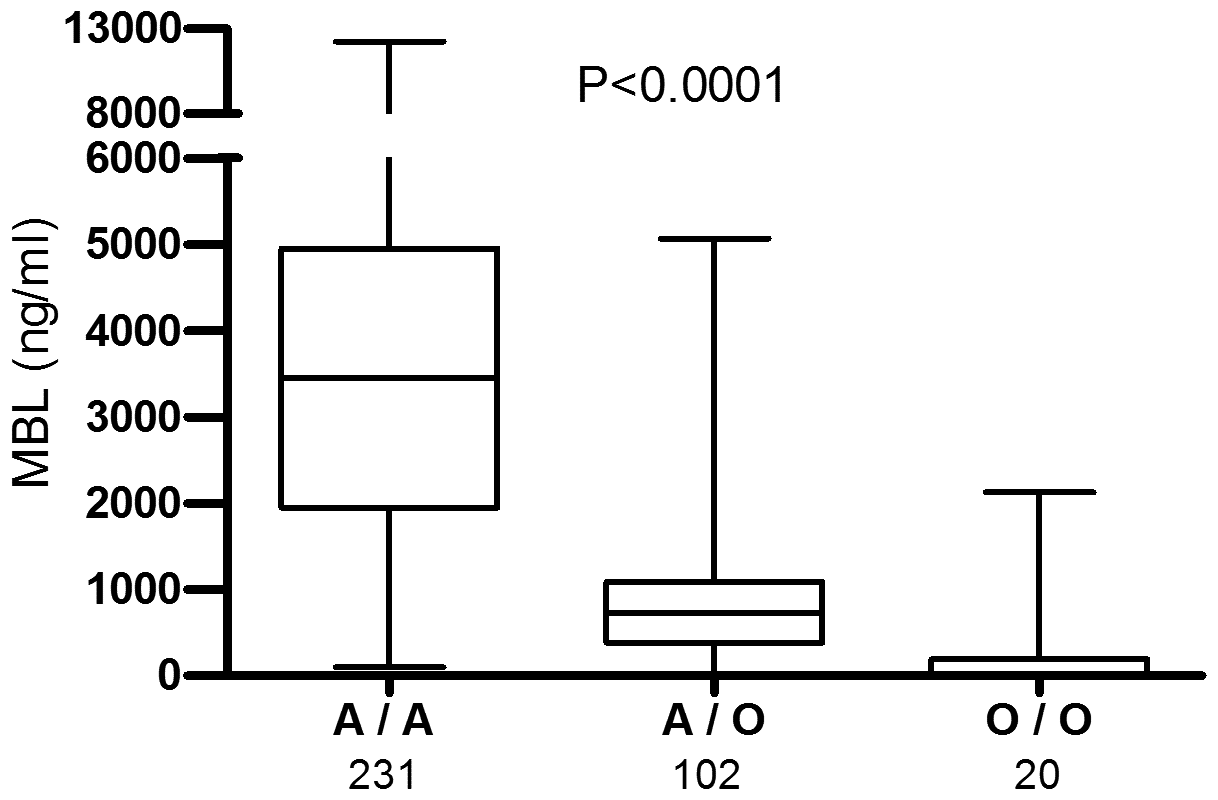
*MBL2* genotypes; A/A, A/O and O/O, with the corresponding MBL concentrations in 355 adolescents (p<0.0001). A/A refers to wild type, A/O heterozygote variants and O/O homozygote variants, and O refers to exon1 variant alleles B, C or D. Statistical significance was calculated with Kruskal-Wallis test. The number of subjects in each group is shown. Data is represented with first and third quartile and median line. The ends of the whiskers represent maximum and minimum values of the data.

**Figure 2:**
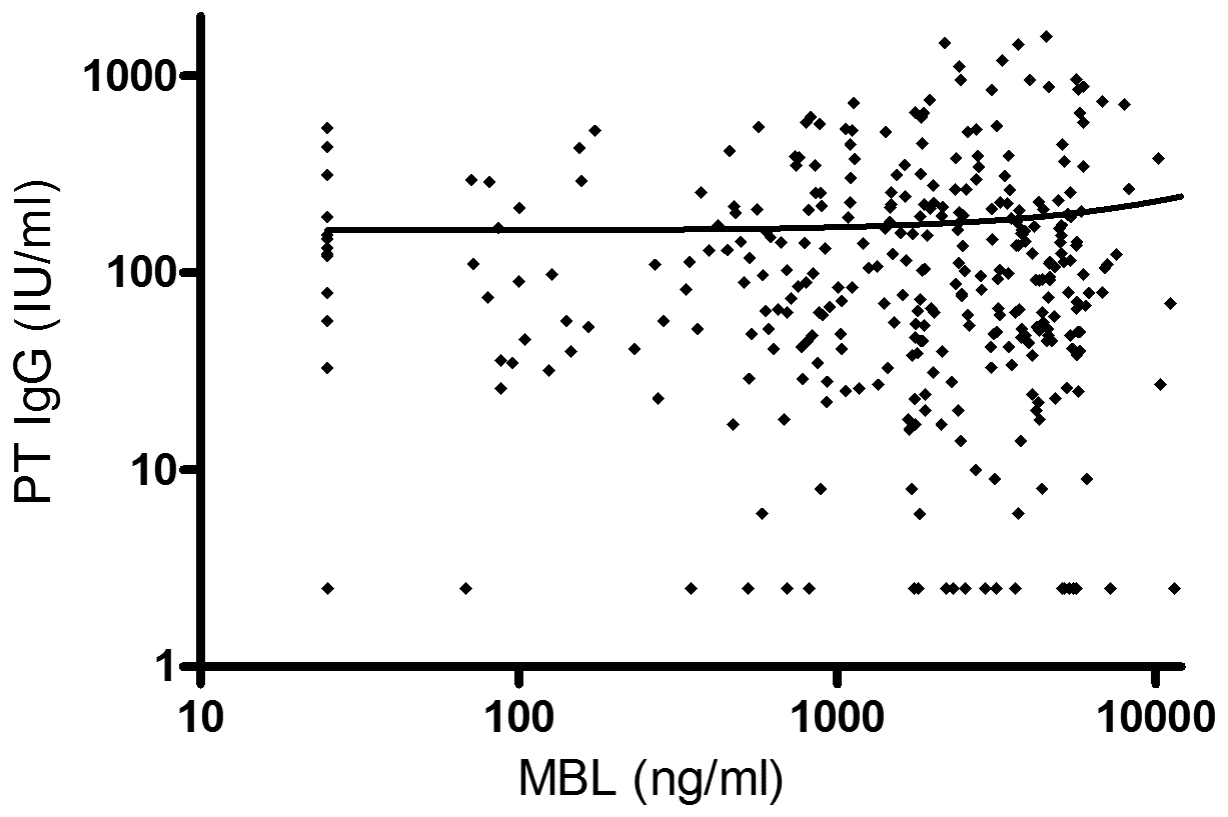
MBL concentrations and PT IgG concentrations after the first booster vaccine in 355 adolescents. Mannose-binding lectin (MBL) concentrations (ng/ml) and IgG antibody concentrations (IU/ml) against pertussis toxin after the first booster vaccine in 355 adolescent subjects. No correlation between MBL and IgG concentrations were observed. Solid line indicates the nonlinear regression curve.

Genotyping was successful in all subjects (355 adolescents and 213 infants). The distribution of MBL genotypes for both cohorts and MBL concentrations of the adolescent cohort are presented in Table 1 and Figure 1. The MBL concentration was significantly higher in subjects with wild type A/A than those with heterozygote variants A/O and with homozygote variants O/O. In subjects with heterozygote variants A/O, the MBL concentration was significantly higher than in those with homozygote variants O/O. There was no difference in the frequencies of *MBL2* genotypes between genders (P = 0.91 and P = 0.67, for adolescent and infant cohort, respectively). No difference was observed in the MBL concentrations between genders in the adolescent group (p = 0.40). There was no correlation between MBL concentration and antibody response against any vaccine antigens in adolescents' cohort after first booster vaccine (for pertussis toxin R^2^ = 0.0004, Figure 2.). In the adolescent cohort, 15 (4.2%) subjects were found to have severe MBL deficiency (<50 ng/ml). As expected, the MBL concentrations are reduced in homozygote variants compared with wild type (Figure 1.) Due to this expected reduction and as no difference was observed in antibody concentrations, MBL concentration was not measured from the infant cohort.

**Table 1:** Frequencies and MBL concentrations for the different *MBL2* genotypes in 355 adolescents and *MBL2* genotype frequencies in 213 infants included in this study.

	Adolescent cohort		Infant cohort
	No of subjects (%)	Median concentration in ng/ml (range)	No of subjects (%)
**A/A**	232 (65.4)	3453 (100–12247)	147 (69.7)
**A/O**	103 (29.0)	725.5 (25–5068)	60 (28.2)
**A/B**	69 (19.4)	584 (25–1251)	40 (18.8)
**A/C**	5 (1.4)	471 (25–835)	1 (0.5)
**A/D**	29 (8.2)	1701 (421–5068)	19 (8.9)
**O/O**	20 (5.6)	25 (25–2128)	4 (1.9)
**B/B**	6 (1.7)	25 (25–593)	2 (0.9)
**B/D**	7 (2.0)	25 (25–81)	0
**D/D**	3 (0.8)	837 (25–2128)	1 (0.5)
**C/D**	1 (0.3)	104.0	0
**C/C**	3 (0.8)	100 (88–852)	0
**C/B**	0	-	1 (0.5)

### MBL genotypes and IgG antibodies to dTpa antigens

In the adolescent cohort, the geometric mean concentration (GMC) of IgG antibodies against all dTpa vaccine antigens were compared between the subjects with MBL wild type A/A and heterozygote variant allele genotypes A/O (A/B, A/C and A/D) and homozygote variant allele genotypes O/O (B/B, B/D, C/D, D/D and C/C) (Table 2.). No significant differences were observed between the genotypes in any time point measured. At ten-year time point, one month after the vaccination, GMC of FHA IgG antibodies differed significantly (p = 0.01) between the AA, AO and OO genotypes. However, there were only three subjects in the OO genotype group. When subjects with severe deficiency of MBL were compared with the rest of the subjects, no significant difference in IgG concentrations against vaccine antigens (diphtheria, tetanus, pertussis toxin, pertacin and filamentous hemagglutinin) were observed.

**Table 2:** Antibody geometric mean concentrations (GMC) after booster doses (pre and post) of acellular pertussis vaccine compained with tetanus and diphtheria toxoidsin 355 adolescent subjects with MBL wild type (A/A), heterozygotes variant type A/O (A/B, A/C, A/D) and homozygote variant type O/O (B/B, B/D, D/D, C/D, C/C).

	MBL A/A		MBL A/O		MBL O/O		P-value*
Timing	n	GMC (95% CI)	n	GMC (95% CI)	n	GMC (95% CI)	
**Pertussis toxin**							
before initial vaccination 10 years ago	227	10.2 (8.4–12.5)	100	10.03 (7.4 – 13.5)	19	7.61 (3.7 – 14.0)	0.59
Month 1	227	85.6 (70.5 – 103.8)	102	82.55 (63.0 – 108.1)	16	62.46 (29.3 – 133.2)	0.74
Year 3	184	15.9 (13.1 – 19.2)	74	17.84 (13.9 – 22.9)	16	16.68 (6.7 – 24.0)	0.50
Year 5	165	12.5 (10.6 – 14.8)	77	12.0 (9.5 – 15.2)	14	8.3 (4.5 –15.3)	0.51
Year 10 prebooster	54	8.3 (0.1 – 11.2)	20	12.6 (7.3 – 22.3)	3	7.1 (0.7 – 68.1)	0.33
Year 10 postbooster	54	90.9 (71.6 – 115.4)	19	88.7 (56.0 – 133.3)	3	131.6 (9.7 – 1788)	0.80
**Pertactin**							
before initial vaccination 10 years ago	230	17.6 (14.3 – 21.7)	103	17.5 (12.6–24.4)	20	16.7 (6.9 – 40.8)	0.97
Month 1	232	386.6 (302.1 – 494.7)	100	412.3 (297.8 – 570.8)	20	326.4 (119.8 – 889.5)	0.94
Year 3	185	113.1 (94.3 – 135.8)	75	96.5 (69.2 – 134.5)	16	148.2 (75.5 – 291.2)	0.43
Year 5	171	84.8 (69.3 – 103.8)	79	64.9 (46.6 – 90.5)	14	62.7 (29.2 – 134.4)	0.28
Year 10 prebooster	54	37.8 (27.4 –52.2)	20	33.8 (17.7 – 64.5)	3	54.8 (2.0 – 1503.0)	0.91
Year 10 postbooster	54	585.8 (466.4 –735.7)	19	450.5 (322.5 – 629.3)	3	1172.0 (293.8 – 4675.0)	0.09
**Filamentous hemagglutinin**							
before initial vaccination 10 years ago	230	63.6 (52.8 – 74.2)	98	65.6 (50.3 85.5)	20	55.0 (27.2 – 111.0)	0.93
Month 1	232	637.0 (533.9 –760.0)	103	667.2 (531.9 – 836.8)	20	623.7 (276.5 – 1407.0)	0.76
Year 3	184	187.8 (166.1 – 212.3)	76	180.9 (150.1 – 218.0)	16	217.9 (140.2 – 338.8)	0.67
Year 5	170	121.8 (106.5 – 137.0)	79	108.0 (87.8 –132.8)	14	173.3 (98.1 – 305.9)	0.12
Year 10 prebooster	54	67.6 (53.8 –84.8)	20	63.0 (46.1 – 86.2)	3	134.9 (47.8 – 380.7)	0.17
Year 10 postbooster	54	865.0 (695.8 – 1075.0)	19	532.7 (412.3 – 688.2)	3	1205.0 (670.2 – 2166.0)	0.01
**Tetanus toxoid**							
before initial vaccination 10 years ago	225	0.6 (0.5 – 0.7)	97	0.5 (0.4 – 0.6)	19	0.6 (0.4 – 0.9)	0.83
Month 1	225	25.9 (23.5 – 28.5)	97	23.0 (19.7 – 26.9)	19	36.5 (27.8 –47.9)	0.06
Year 3	185	2.5 (2.2 – 2.8)	76	2.1 (1.8 – 2.6)	16	3.3 (2.5 – 4.4)	0.08
Year 5	171	2.0 (1.8 – 2.3)	78	2.0 (1.6 – 2.3)	14	2.1 (1.5 – 3.0)	0.92
Year 10 prebooster	54	1.2 (0.8 – 1.7)	20	1.2 (0.8 – 1.8)	3	1.1 (0.2 – 5.8)	0.99
Year 10 postbooster	54	10.1 (8.4 – 12.1)	19	7.8 (4.6 – 13.1)	3	14.1 (4.6 – 42.7)	0.43
**Diphtheria toxin**							
before initial vaccination 10 years ago	223	0.2 (0.2 – 0.2)	97	0.2 (0.2 – 0.3)	19	0.2 (0.1 – 0.3)	0.54
Month 1	225	7.2 (6.2 – 8.2)	98	7.4 (6.0 – 9.0)	19	8.5 (5.0 – 14.2)	0.87
Year 3	184	0.5 (0.5 – 0.6)	76	0.6 (0.5 – 0.8)	16	0.6 (0.3 – 1.0)	0.92
Year 5	171	0.4 (0.3 – 0.5)	78	0.5 (0.4 – 0.7)	14	0.5 (0.3 – 1.0)	0.32
Year 10 prebooster	54	0.3 (0.2 – 0.4)	20	0.3 (0.2 – 0.6)	3	0.5 (0.1 – 2.3)	0.67
Year 10 postbooster	54	5.5 (4.1 – 7.5)	19	5.1 (3.6 – 7.3)	3	17.3 (0.7 – 451.5)	0.26

Abbreviations: GMC, geometric mean concentration; CI, confidence interval; *Kruskal-Wallis test for comparison of antibody concentrations between genotypes A/A, A/O and O/O. P-value <0.05 is considered as statistically significant.

Similarly, in the infant cohort, the GMC of IgG antibodies against PT, PRN and FHA were compared between the subjects with MBL wild type A/A, A/O genotypes (A/B, A/C and A/D) and O/O genotypes (B/B, D/D and C/B) at 2,5 months, 13 months and 2 year time point (Table 3.). No significant differences in PT, PRN or FHA IgG concentrations were observed between the different MBL genotypes, in any time point measured.

**Table 3:** Geometric mean concentrations (GMC) of IgG antibodies against pertussis toxin, filamentous hemagglutinin and pertactin in 213 infant subjects with MBL wild type (A/A), heterozygotes variant type A/O (A/B, A/C, A/D) and homozygote variant type O/O (B/B, D/D, C/B) at age of 2,5 months, 13 months and 2 years.

	MBL A/A		MBL A/O		MBL O/O		P-value*
Pertussis toxin	n	GMC (95% CI)	n	GMC (95% CI)	n	GMC (95% CI)	
2.5 months	45	0.8 (0.5 – 1.2)	24	1.1 (0.6 – 2.1)	3	1.1 (0.6 – 2.1)	0.39
13 months	147	50.7 (43.4 – 59.2)	60	42.2 (29.2 – 60.9)	4	69.8 (37.0 – 131.6)	0.75
2 years	78	6.8 (5.3 – 8.6)	28	6.8 (4.2 – 11.1)	2	4.9 (0.4 – 64.4)	0.71
**Pertactin**							
2.5 months	45	1.8 (1.1 – 3.2)	24	2.6 (1.5 – 4.5)	3	2.0 (0.1 – 38.7)	0.73
13 months	147	38.5 (26.3 – 56.4)	60	26.9 (14.2 – 50.8)	4	70.0 (8.1 – 604.4)	0.48
2 years	78	7.1 (4.8 – 10.5)	28	7.7 (4.2 – 14.5)	2	16.3 (7.5 – 35.5)	0.78
**Filamentous hemagglutinin**							
2.5 months	45	1.8 (1.0 – 3.3)	24	3.6 (2.0 – 6.7)	3	2.2 (0.3 – 16.0)	0.43
13 months	147	164.4 (142.7 – 189.4)	60	145.7 (102.0 – 208.0)	4	203.6 (135.1 – 306.9)	0.88
2 years	78	32.6 (26.9 – 39.5)	28	36.4 (24.1 – 54.7)	2	12.0 (12.0 – 12.0)	0.19

Abbreviations: GMC, geometric mean concentration; CI, confidence interval; *Kruskal-Wallis test for comparison of antibody concentrations between genotypes A/A, A/O and O/O. P-value <0.05 is considered as statistically significant.

## Discussion

A proper antibody response after vaccination is important and the high level of antibodies might be implicated in good protection [6], [7]. In humans, polymorphism in the *MBL2* gene has been previously shown to affect immune reactions to influenza vaccine [14]. Our previous study indicated that MBL deficiency increases the risk for pertussis in adults [19]. In this study, the perspective was enlarged to the pertussis vaccine responses. No such studies have been conducted previously in humans. Our two cohorts, composed of adolescents and infants, enabled us to investigate comprehensively the possible association of *MBL2* polymorphisms in exon 1 with the production and persistence of IgG antibodies both after dTpa booster vaccination and after dTpa primary vaccination. The adolescent cohort is part of the 10-year follow-up study performed in Finland, in which adolescents 11–13 years of age received acellular pertussis booster vaccine and a secondary booster ten years after [21]–[24]. Infant cohort is a subgroup from a large prospective cohort study performed in Finland [25]. To our knowledge, our study is the first to investigate possible association between acellular pertussis vaccine responses and MBL polymorphisms. The frequency of *MBL2* polymorphisms in codons 52, 54 and 57 observed in this study were almost identical to others conducted in Finnish population [26], [31] and other European populations [32].

Similarly to previous reports [33], [34], also in our study the MBL genotypes reflect the serum concentration of MBL. Individuals in the adolescent cohort with wild type MBL had the highest concentration, whereas those with homozygote variant allele genotypes had the lowest concentration of MBL. The deficiency of MBL, caused by the polymorphisms in *MBL2* gene, has been associated with susceptibility to various infectious diseases with wide etiology from both virus and bacteria [20]. It has not been previously studied, whether deficient concentration of MBL in serum is related to antibody responses or persistence of antibodies after pertussis vaccinations. MBL concentration measurement from the adolescent cohort enabled us to study this aspect. However, we did not observe any differences in antibody production between the subjects with total deficiency of MBL (<50 ng/ml) compared with the rest of the subjects. Our definition, used also in our previous study [19], is though more stringent than 500 ng/ml, which Eisen et al. has proposed to be used [35]. To further prove this negative finding, we looked in the adolescent cohort whether the MBL concentration correlates with IgG antibody response against vaccine antigens after first booster vaccination. No correlation was observed (pertussis toxin, Figure 2.). This lack of association in MBL serum concentration with antibody responses after booster pertussis vaccination concluded us not to further test the infant cohort for MBL deficiency.

Immature immune system in infants combined with unexpected low response to vaccine can cause vulnerability for infectious diseases. Equivalent in adolescents, failure in booster vaccine responses decrease the level of protection. Generally, the role of innate immunity after both primary and booster pertussis vaccination is mainly unknown. Strength of our study is the large number of subjects in two different age groups with serial follow-up, which allows us to enlighten the role of innate immunity in vaccine responses after dTap vaccinations. However, results from our study cohorts indicate that in both age groups *MBL2* gene polymorphism does not affect dTpa vaccine responses or persistence of antibodies. Only significant difference observed was in the adolescent cohort with FHA antibodies at one time point. Subjects with MBL O/O genotype had the highest GMC of IgG against FHA after second booster. The result is though based on only three individuals.

Classically, MBL has been defined as a pattern recognition molecule of the innate immune system, which is able to bind many microorganisms with its carbohydrate recognition domains [13]. However, more recent works have extended its role to conditions such as the recognition of apoptotic cells and modulation of inflammation [34]. So far, studies concerning MBL and vaccine-induced responses are very limited. In humans, to our knowledge, only one study concerning the role of MBL with altered response to vaccine has been reported [14]. Tang et al reported that the *MBL2* polymorphism in codon 54 (variant B) caused a decreased risk for developing a poor antibody response after influenza vaccination compared with the normal responders [14]. In our study, we did not find such an association. The contrary finding between our study and Tang's may be due the different vaccine used in these two studies: inactivated influenza A vaccine in their study, whereas ours is related to bacterial vaccine based on purified proteins. Differing recognition of antigens might affect as well.

Other studies on vaccine responses and MBL polymorphism are only reported in animal models [16], [15]. The study by Guttormsen et al. [16] found that MBL null mice produced higher IgG concentration against tetanus toxoid vaccine after immunization compared with wild type mice, proposing that MBL might inhibit the IgG production after vaccination. Similar finding has been reported with chickens [15]. In this study by Juul-Madsen et al. [15], chickens were vaccinated against infectious bronchitis virus (IBV) with or without addition of mannose to the vaccine. They found that when mannose was administrated together with the IBV vaccine, chickens with the low production of MBL produced IBV specific antibodies significantly more than chickens with high MBL level. However, it has been shown with mouse model immunized with hepatitis B surface antigen that the modulation of antibody responses by MBL depends on the genetic environment [36]. Furthermore, it might be more complex to compare the vaccine responses in humans with animal models.

It has not been shown whether MBL is able to recognize *B. pertussis* or its surface molecules. Our previous study indicated that MBL deficiency might increase the risk of pertussis in adolescents [19]. This contrary finding might be caused by the fact that acellular pertussis vaccine used in Finland is composed of three pertussis antigens, pertactin, pertussis toxin and filamentous hemagglutinin, and it is not known whether MBL recognizes these antigens. In addition, acellular vaccine does not include carbohydrate components such as lipopolysaccharides, the well-known epitopes of MBL recognition [37]. On the surface of *B. pertussis* there are though more of potential epitopes for MBL to recognize. The role of MBL in the activation of immune responses might differ between infection and vaccination.

We acknowledge the limitations of our study. The number of male and female subjects in the adolescent cohort is slightly unbalanced. This is because a part of the male subjects were ineligible to participate the 10-year follow-up, due to the diphtheria and tetanus booster vaccine they had received during the national military service [24], though this unbalance did not affect the frequency of *MBL2* exon1 SNPs observed. Secondly, we did not study the polymorphism detected in the promoter region of the MBL. However, the three SNPs in exon1 included in this study are the ones that cause decrease in MBL level by approximately 90% [32].

In conclusion, this study indicates that *MBL2* polymorphisms in exon 1 and deficient concentration of MBL in serum are not associated with the production and the persistence of antibodies after primary and booster acellular pertussis vaccination. Our finding also suggests that MBL may not be involved in modulating antibody responses to vaccines made of purified bacterial proteins in humans.
